# Adverse early-life conditions and their impact on the marriage market and social mobility: The Poznań birth cohort, 1830–1855

**DOI:** 10.1371/journal.pone.0345394

**Published:** 2026-09-28

**Authors:** Szymon Antosik, Tommy Bengtsson, Piotr Antosik, Grażyna Liczbińska

**Affiliations:** 1 Doctoral School of Humanities, Adam Mickiewicz University, Poznań, Poland; 2 Centre for Economic Demography and Department of Economic History, Lund University, Lund, Sweden; 3 Independent researcher, Kielce, Poland; 4 Institute of Human Biology and Evolution, Faculty of Biology, Adam Mickiewicz University, Poznań, Poland; Anhui University of Chinese Medicine, CHINA

## Abstract

**Objectives:**

Early-life conditions may influence later-life outcomes through biological scarring and selective survival mechanisms. However, the long-term effects of childhood adversity on marriage formation and social mobility remain insufficiently explored in historical populations. This study examines whether exposure to adverse early-life conditions affected the probability of entering a first marriage and social mobility at first marriage in 19th-century Poznań.

**Methods:**

The study draws on linked individual-level data from the Poznań Historical Population Database, including birth, marriage and death records from the Catholic and Protestant parishes of Poznań. The analysis focuses on individuals born between 1830 and 1855 who survived at least age 15. Logistic regression models were used to examine (1) the association between early-life adversity and the probability of entering first marriage by age 45 (N = 15,121), and (2) the relationship between early-life conditions and social mobility between birth and first marriage among individuals whose first marriage occurred by age 45 and for whom socioeconomic position at birth and marriage could be determined (N = 802). Early-life adversity was assessed using population-level indicators of prenatal, infant, and childhood conditions, including the sex ratio at birth, floods, rye prices, infant mortality rate, and crude death rate.

**Results:**

A low sex ratio at birth in the year of conception and high rye prices during infancy, both indicative of adverse conditions, were associated with lower odds of entering marriage. In contrast, high rye prices during the prenatal period were associated with a higher probability of marriage, which may reflect selective survival processes. Exposure to high infant mortality did not significantly affect the probability of marriage. Analyses of social mobility revealed limited effects of early-life adversity among individuals who survived and entered marriage. However, exposure to high crude death rates at ages two and three was associated with lower odds of downward mobility.

**Conclusion:**

Early-life adversity influenced later-life demographic outcomes in 19th-century Poznań, particularly marriage formation. The findings suggest that both scarring and selection mechanisms shaped the observed associations between childhood conditions and adult outcomes. The limited effects on social mobility highlight the importance of considering selective survival and the restricted nature of historical marriage records when analysing the long-term consequences of adverse early-life conditions.

## Introduction

### Early-life conditions and later-life socioeconomic outcomes

Exposure to adverse conditions during early life may have long-lasting consequences for health and socioeconomic attainment. The pioneering studies of Forsdahl [1] and Barker [2–4] established the developmental origins perspective, demonstrating that the socioeconomic environment experienced during the sensitive period of development in the first year of life can influence health trajectories across the life course. Poor health, in turn, may contribute to lower educational achievement, reduced occupational opportunities, and low socioeconomic status. Evidence from both historical and contemporary populations supports these relationships. Using 19th-century data from rural southern Sweden, Bengtsson and Broström [5] showed that disease exposure during the first year of life negatively affected socioeconomic position at the age of fifty. Similarly, Quaranta [6], analysing women born between 1813 and 1898 in the same region of Scania, demonstrated that individuals originating from lower social strata and born in years characterised by high infant mortality or during whooping cough epidemics faced reduced chances of attaining a high socioeconomic status in adulthood. At lower ages, up to about 25 years of age, scarring and selection cancel out. Comparable findings have been reported for modern populations. Analysing the 1958 British birth cohort, Palloni et al. [7] found that poor early-life health was associated with lower adult socioeconomic position, partly through its effects on cognitive development and educational attainment. These findings are consistent with broader evidence indicating that conditions experienced early in life contribute substantially to the reproduction of socioeconomic inequalities [8,9]. As Heckman observed, “a large literature documents the importance of the early years in determining adult capabilities of cognition, motivation, and health” [10]. Thus, adverse conditions experienced during sensitive developmental periods may influence not only biological outcomes but also later social opportunities, including educational attainment or occupational trajectories [11–13].

Historical populations provide a particularly valuable setting for examining these mechanisms because they experienced substantial variation in living conditions, nutritional resources, and disease environments. Previous research has shown that early-life nutritional deprivation has long-term consequences for cognitive abilities and socioeconomic attainment. A study of the historical population of the United Kingdom at the turn of the eighteenth and nineteenth centuries demonstrated that individuals born during periods of high food prices exhibited poorer cognitive performance and were more likely to enter lower-paid occupations in adulthood [14]. Similar patterns have been observed in twentieth-century Poland, where indicators of physical development were positively associated with educational achievement [15]. These findings suggest that conditions experienced during sensitive developmental periods may influence later-life capabilities through interconnected biological, cognitive, and socioeconomic pathways.

### Measuring early-life adversity in historical populations

An important challenge in historical demography is the measurement of prenatal and early-life conditions, as direct information on maternal health, fetal development, household nutrition, and childhood disease exposure is rarely available. Consequently, researchers have relied on population-level indicators that capture variation in environmental conditions during sensitive stages of development [5,6,16].

One indicator increasingly used to assess prenatal conditions is the sex ratio at birth (SRB), defined as the proportion of male to female live births. The biological rationale for using SRB as an indicator of prenatal adversity is based on the greater vulnerability of male embryos and fetuses to adverse intrauterine environments. Maternal nutritional deprivation, infectious diseases, psychological stress, and other forms of environmental hardship may increase male fetal losses disproportionately compared with female fetuses, resulting in temporary declines in the proportion of male births. Consequently, unusually low values of SRB may be interpreted as indirect indicators of deteriorating prenatal environments and increased maternal stress. Importantly, SRB does not measure individual maternal or fetal conditions directly but rather captures changes in reproductive outcomes occurring at the population level. Historical studies have documented substantial fluctuations in SRB during periods of famine, epidemics, war, and socioeconomic crises, supporting its use as a proxy for prenatal adversity [17–24]. In addition to prenatal indicators, historical demographers have employed measures reflecting other dimensions of early-life environments, including infant and childhood mortality and fluctuations in food prices [16,25–27]. These indicators capture different, although partly overlapping, dimensions of early-life adversity, including disease exposure, nutritional conditions, and household economic stress.

Another quite novel indicator is potential exposure to natural disasters, such as floods. Numerous studies indicate that maternal stress resulting from floods or other natural disasters can have long-term negative effects on children [28–30]. A study by Willführ and Perez on the 1825 flood in the Krummhörn region of eastern Frisia in Germany found that men who experienced the flood before age 5 were more likely to remain unmarried, whereas women who were teenagers at the time of the flood married later than average [31]. A similar mechanism is indicated by Rosales-Rueda [32], who analysed the long-term effects of exposure to floods induced by El Niño in Ecuador in 1997–1998 on human capital formation. Children exposed prenatally were shorter and scored lower on cognitive tests several years later. The author suggests that possible causes include adverse perinatal conditions, such as low birth weight, particularly when exposure occurs in the third trimester, and a reduction in household resources following the disaster, which could limit investment in children.

### Early-life adversity, marriage formation, and social mobility in historical populations

Beyond health and occupational attainment, marriage represents another important adult outcome that may be shaped by early-life conditions and subsequent health trajectories. In historical societies, marriage was not only a personal relationship but also a fundamental social and economic institution that determined access to household formation, economic cooperation, and social reproduction [33]. Consequently, factors influencing the probability of marriage provide important insights into how early-life environments affected later-life social outcomes. Marriage markets were shaped by both socioeconomic resources and characteristics associated with health and physical condition. Individuals were evaluated not only according to family background and economic prospects but also according to traits interpreted as indicators of vitality, fertility, and the ability to contribute to household formation. Adverse conditions experienced during early life could therefore influence marriage outcomes through both biological pathways, by affecting health and physical development, and social pathways, by shaping perceptions of suitability as a spouse.

Studies of the Polish population indicate that in 19th-century Poznań, male marriage choices prioritised physical attractiveness and youth over the social status of potential partners, whereas women placed greater importance on the socioeconomic position of prospective husbands [34,35]. Similar associations between health-related characteristics and marriage opportunities have been documented in other historical populations [36–44]. These studies suggest that health and physical condition influenced partner selection and life-course trajectories in historical societies. However, previous research has largely focused on direct indicators of health, such as stature, visible disease markers, or infant and child mortality within a family. Less is known about whether broader environmental conditions experienced during the prenatal period, infancy, and early childhood affected later access to marriage.

Marriage also represented a potential pathway of social mobility. In historical populations, social status could change either through an individual’s occupational career or through marriage [45]. Entering marriage was not random but depended on survival, health, socioeconomic resources, and social circumstances. Especially in large cities, a substantial share of individuals remained unmarried at ages 45–54, a phenomenon known as definitive celibacy and commonly interpreted as an indicator of permanent non-marriage [46–48]. In eighteenth- and nineteenth-century Europe, on average, around 10 per cent of the population lived in definitive celibacy, while in some communities this share reached as much as 38 per cent [49]. Marriage and household formation were therefore closely connected with the availability of resources, particularly parental wealth and social position [50,51]. At the same time, marriage itself was associated with better health and economic outcomes because married individuals generally benefited from spousal support and, later, support from children [52]. This interaction between early-life conditions, marriage selection, and socioeconomic attainment suggests that adverse conditions may operate through two distinct mechanisms. First, they may reduce the probability of entering marriage by affecting health, survival, or social attractiveness. Second, among those who marry, early-life conditions may influence the socioeconomic position achieved in adulthood. Distinguishing between these processes is therefore essential for understanding the long-term social consequences of early-life adversity in historical populations.

### Study aims and research questions

Despite extensive research on the long-term consequences of early-life adversity, less is known about how prenatal and childhood conditions influenced marriage formation and socioeconomic mobility in historical urban populations. Previous studies have demonstrated associations between early-life environments and adult health, cognitive abilities, mortality, and occupational outcomes; however, marriage as a potential pathway linking early-life conditions with later social position remains insufficiently explored. 19th-century Poznań provides an important setting for examining these mechanisms. The city experienced substantial population growth, social heterogeneity, recurrent infectious disease outbreaks, and fluctuations in food availability [53–55]. At the same time, detailed parish records allow the reconstruction of individual life courses and the investigation of long-term consequences of early-life conditions.

This article examines whether adverse early-life conditions influenced later demographic and socioeconomic outcomes, considering both selection into marriage and socioeconomic attainment among those who married. We address two interrelated research questions:

(1) Did exposure to adverse conditions during early life reduce the likelihood of entering a first marriage in 19th-century Poznań, reflecting possible health-related selection into marriage?
(2) Among individuals who entered marriage, did exposure to early-life adversity influence socioeconomic mobility at the time of first marriage, as reflected in differences between parental socioeconomic status and individual occupational position?

By distinguishing between these processes, this study contributes to understanding how early-life environments shaped later-life trajectories in a historical urban population.

## Materials and methods

### Study population and sample selection

The study uses individual-level data from the Poznań Historical Population Database, which contains demographic records from five Catholic and one Protestant parishes operating in 19th-century Poznań [56]. We focus on individuals born between 1830 and 1855 and follow their life histories until their first marriage, censoring at age 45. The upper age limit was selected to capture first-marriage behaviour while reducing the influence of very late marriages and permanent non-marriage. Only individuals who survived to at least age 15 were included, as this was the minimum age for entering marriage. Given high infant and childhood mortality, this restriction ensures that the analytical sample consists of individuals who could realistically experience marriage and subsequent socioeconomic mobility. After applying these criteria, the analytical dataset for the analysis of marriage formation comprised 15,121 individuals. This sample was used in Model 1, which estimates the probability of entering a first marriage by age 45. Married individuals are not a random subset of the population, as marriage was socially and economically selective. Ignoring the selective nature of marriage would bias analyses of social mobility [57].

To analyse socioeconomic mobility, we constructed a second analytical sample consisting only of individuals whose birth and marriage records could be successfully linked and for whom occupational information was available. Occupational information for the individuals in our sample is available only for those who married, as it is recorded in their marriage certificates. The birth records of any children born to these individuals may also contain information about the individuals’ occupations as parents, but these records have not yet been linked to the dataset used in this study. We therefore use parental socioeconomic status at the time of each individual’s birth as a measure of initial social position and compare it with the individual’s own socioeconomic status at the time of marriage. This sample was used in Model 2, which examines changes in socioeconomic position between parental status at birth and occupational status recorded at first marriage.

A marriage record was identified for 905 individuals who entered their first marriage by age 45. After additionally restricting the sample to individuals with occupational information available both at birth and at marriage, the final analytical sample for Model 2 comprised 802 individuals.

Individual records were linked using family reconstitution and direct matching between birth and marriage certificates. The process was automated in Python and manually verified to eliminate false matches. Linkage relied on name similarity using Levenshtein distance, with blocking by age, sex, event date, and female reproductive age constraints [58,59].

### Social structure and occupational classification

Occupational information was used to reconstruct socioeconomic position at birth and at first marriage. Because occupations represented an important dimension of social status in nineteenth-century urban populations, occupational data were analysed using historical classification systems. To provide contextual information on socioeconomic differentiation within 19th-century Poznań, we used occupational data of inhabitants across five urban quarters based on the Poznań address book published in 1844 [60]. Address books, also known as city directories, have been used quite rarely in research so far, although they are a valuable source of individual-level data [61]. Occupations derived from the address book were classified using HISCAM scores (historical CAMSIS), a continuous measure of social status [62]. HISCAM scores were calculated for five urban quarters in 1844, illustrating spatial differences in the socioeconomic composition of the city (Fig 1). Higher HISCAM values indicate occupations associated with higher social standing. Fig 1 shows that socioeconomic differentiation was not evenly distributed across urban space: some quarters contained a greater concentration of higher-status occupations, whereas others were characterised by occupations associated with lower social standing. The figure provides contextual evidence that occupational categories used in this study reflect meaningful social differences within 19th-century Poznań rather than merely administrative or occupational distinctions. For the analysis of intergenerational mobility, occupational and status information was classified using the Social Power (SOCPO) scheme [63]. SOCPO was selected because it provides historically meaningful categories that enable comparisons between parental social position and individual occupational attainment across generations. It was also better suited to our study sample than the commonly used HISCLASS (Historical International Social Class Scheme) [64], because HISCLASS was developed primarily for classifying specific occupations and employment relations. Many records in our dataset did not refer to a clearly defined occupation but instead described livelihoods based on capital ownership or indicated social or legal status, such as burgher. Such entries cannot be consistently classified using HISCLASS, which relies on occupational information as the primary basis for categorisation. SOCPO therefore offered a more appropriate framework for capturing the broader dimensions of historical social position represented in our sources.

**Fig 1:**
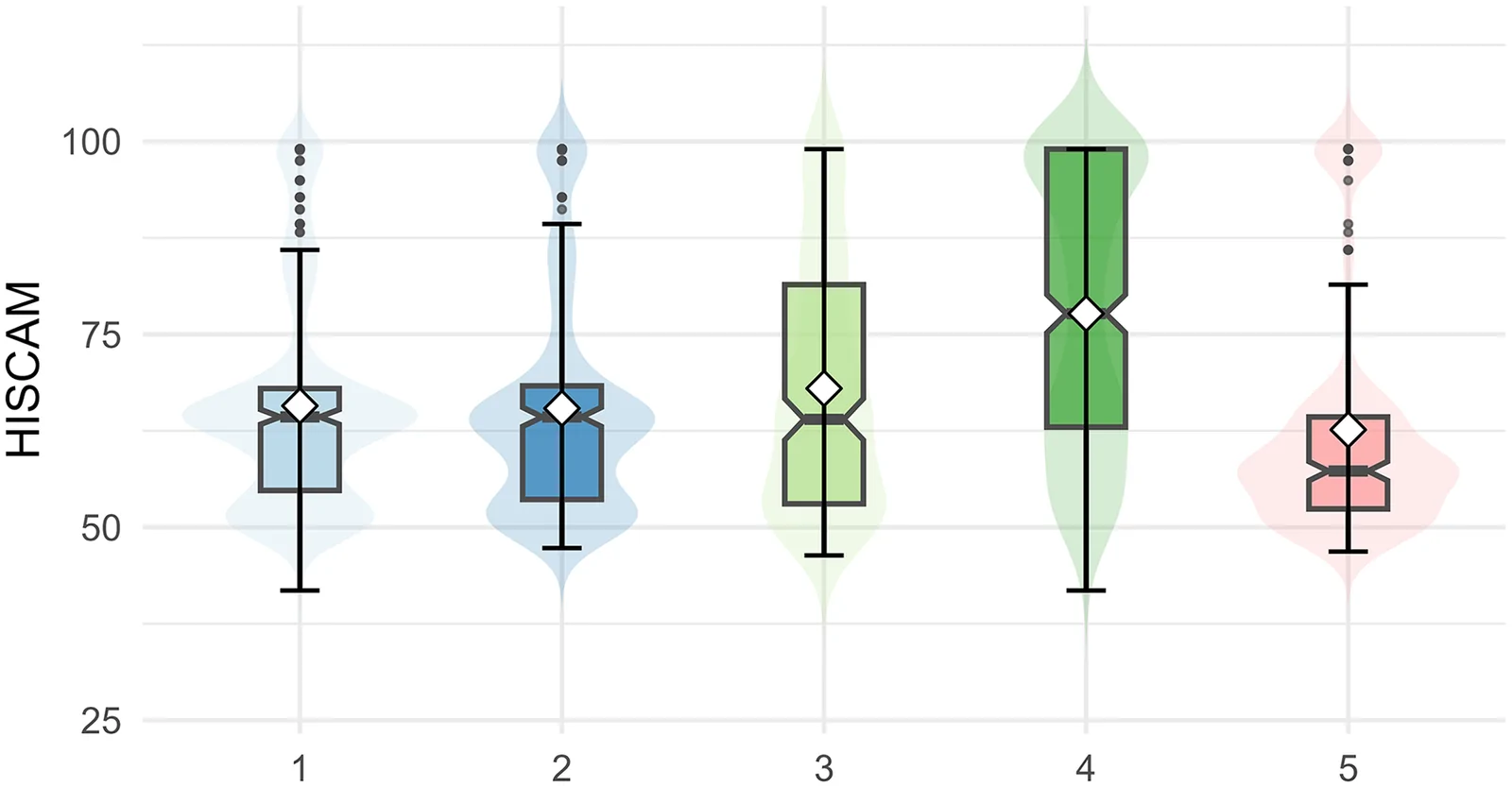
Socioeconomic differentiation across the five urban districts of Poznań in 1844 based on HISCAM scores. Source: Authors’ calculations based on [60]. Numbers 1–5 indicate the city districts.

### Indicators of early-life adverse conditions

We examined exposure to adverse conditions during three critical stages of early life: the prenatal period, infancy (the first year of life), and early childhood (the second and third years of life). The selected indicators were chosen to capture different dimensions of early-life environments, including prenatal stress, disease burden, and nutritional conditions.

For the prenatal period, we used the sex ratio at birth (SRB), calculated as the ratio of male to female live births in a given year and assigned to individuals according to their year of conception. In addition, prenatal exposure to environmental stress was measured using the occurrence of floods during pregnancy. Infant mortality rate (IMR) was used as an indicator of conditions during the first year of life. For exposure during the second and third years of life, we used the crude death rate (CDR), which reflects population-level mortality conditions in early childhood.

The price of rye was used as an indicator of food availability and economic stress during each critical period. Rye was selected because it was a major staple food in 19th-century Central Europe and price fluctuations reflected changes in the availability and affordability of basic food resources. Demographic indicators (SRB, IMR, and CDR) were calculated using data from the Poznań Historical Population Database. Flood exposure was identified using historical measurements of the Warta River water level. Information on rye prices was obtained from contemporary official publications. Individuals were classified as exposed to adverse early-life conditions when at least one observation during the relevant developmental period was identified as an extreme deviation from the expected level of the respective indicator. The procedure used to identify such deviations is described below.

### Construction of exposure indicators

To identify periods of unusually adverse conditions, we used a deviation-based approach adapted from procedures previously applied to study mortality crises in nineteenth-century Denmark [65]. The aim was to identify years or months characterised by extreme values of the selected indicators rather than to classify absolute levels of mortality, prices, or environmental conditions.

For infant mortality rate, crude death rate, and rye prices, monthly values were analysed. The reference values for each indicator were calculated separately for each observation using observations from the five preceding and five following years relative to the observation of interest. Because periods of crisis could influence the calculation of reference values, the procedure included iterative removal of extreme observations. Values deviating by more than three standard deviations from the preliminary mean were excluded, and the mean and standard deviation were recalculated until no further extreme values remained. The observed values were then compared with the corresponding reference values. Observations exceeding the reference mean by three standard deviations were classified as extreme events.

For the sex ratio at birth, annual values were used because monthly estimates exhibited excessive variability due to relatively small numbers of births. For SRB, only negative deviations were considered adverse, because unusually low proportions of male births may indicate increased prenatal loss of male fetuses. For mortality indicators and rye prices, positive deviations were treated as adverse because they reflected excess mortality or increased economic pressure. Results of extreme observations for SRB, IMR and CDR are shown in Figs 2–4.

**Fig 2:**
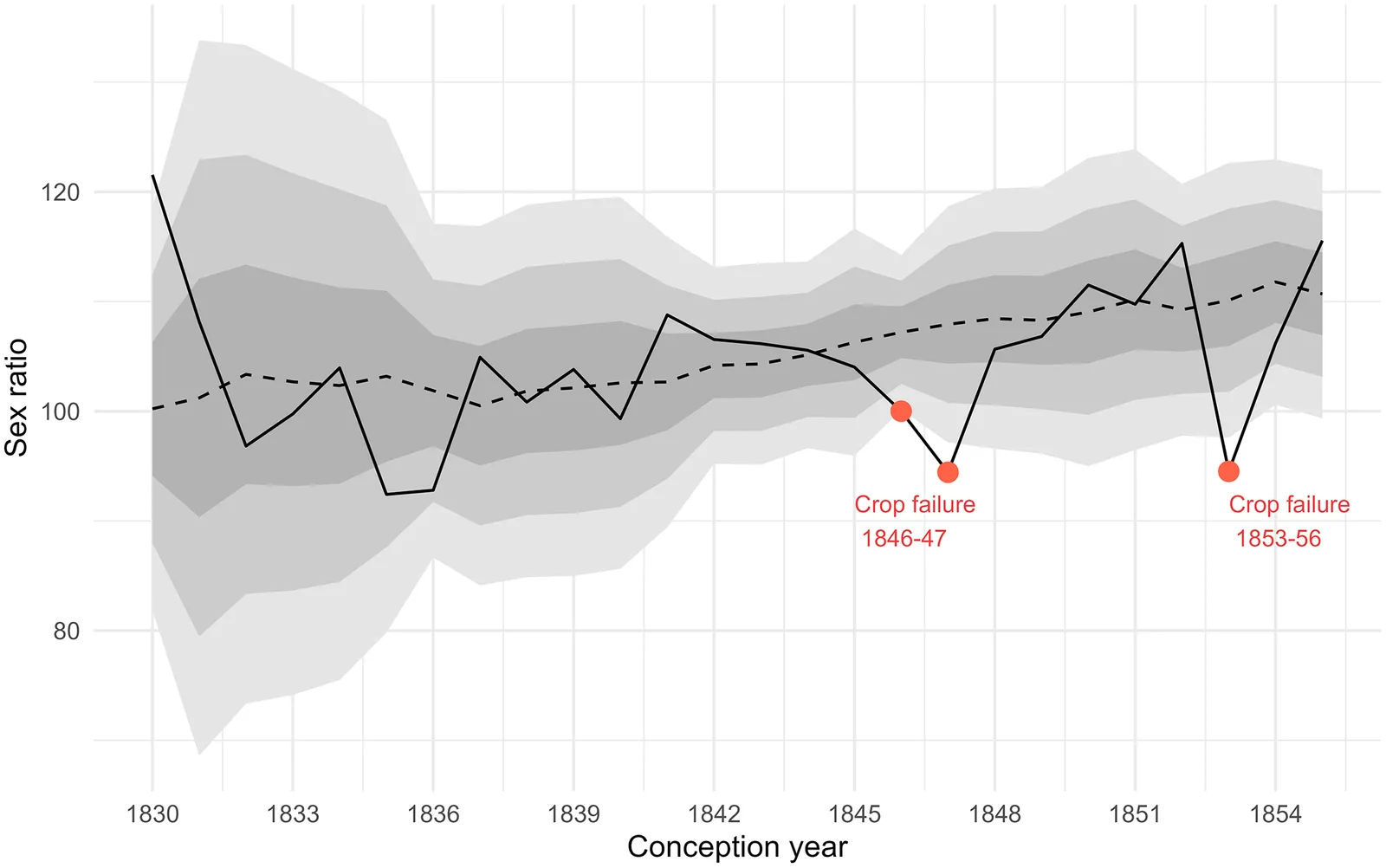
Annual sex ratio at birth in Poznań and years with unusually low values, 1830–1855. Authors’ calculations based on the Poznań Historical Population Database. The dashed line indicates the reference mean, the solid line represents annual values, and the grey shaded area denotes the ± 3 standard deviation range. Outliers are highlighted in red.

**Fig 3:**
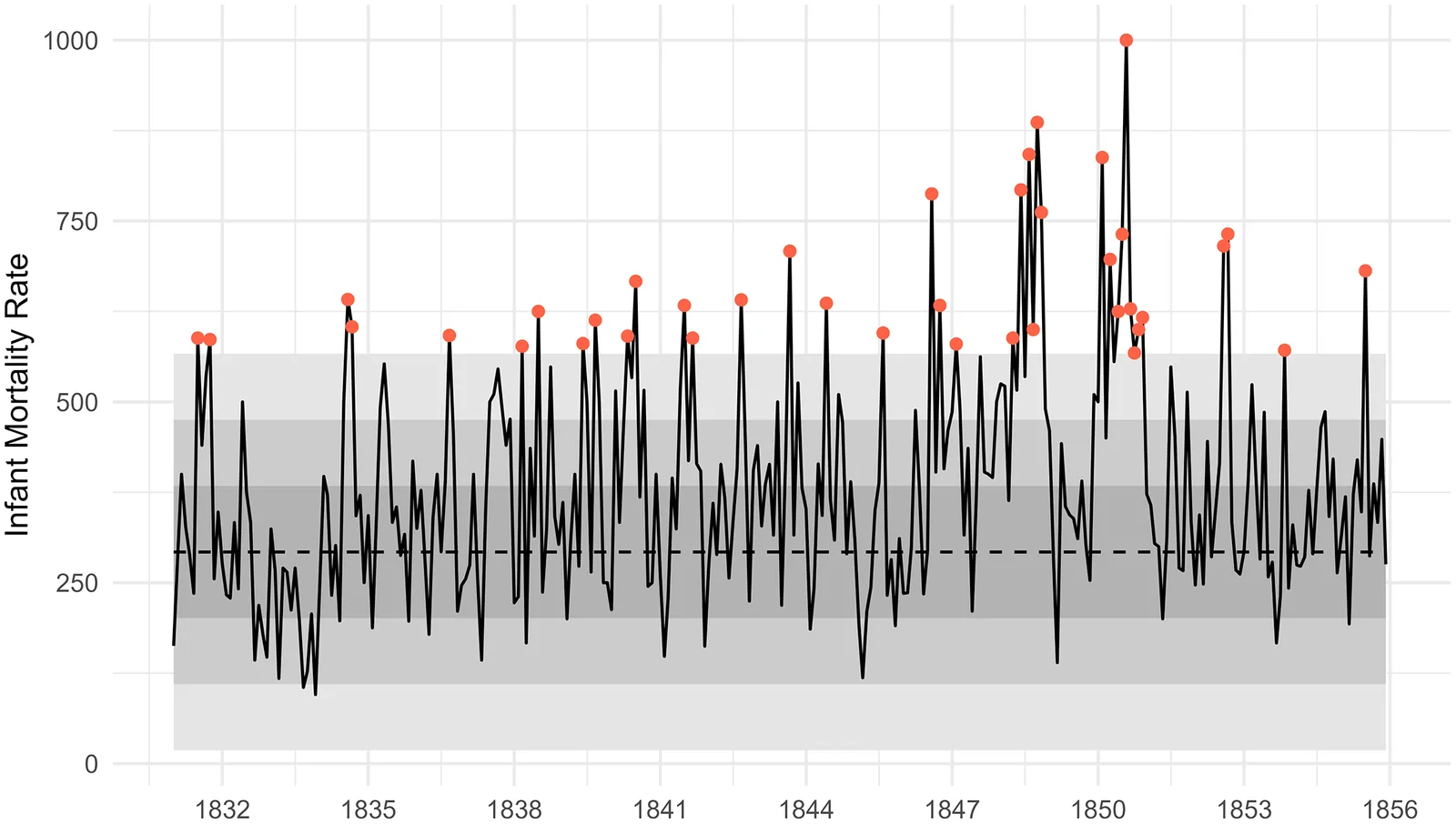
Monthly infant mortality rate in Poznań and observations with unusually high values, 1831–1855. Authors’ calculations based on the Poznań Historical Population Database. The dashed line indicates the reference mean, the solid line represents monthly infant mortality rates, and the grey shaded area denotes the ± 3 standard deviation range. Outliers are highlighted in red.

**Fig 4:**
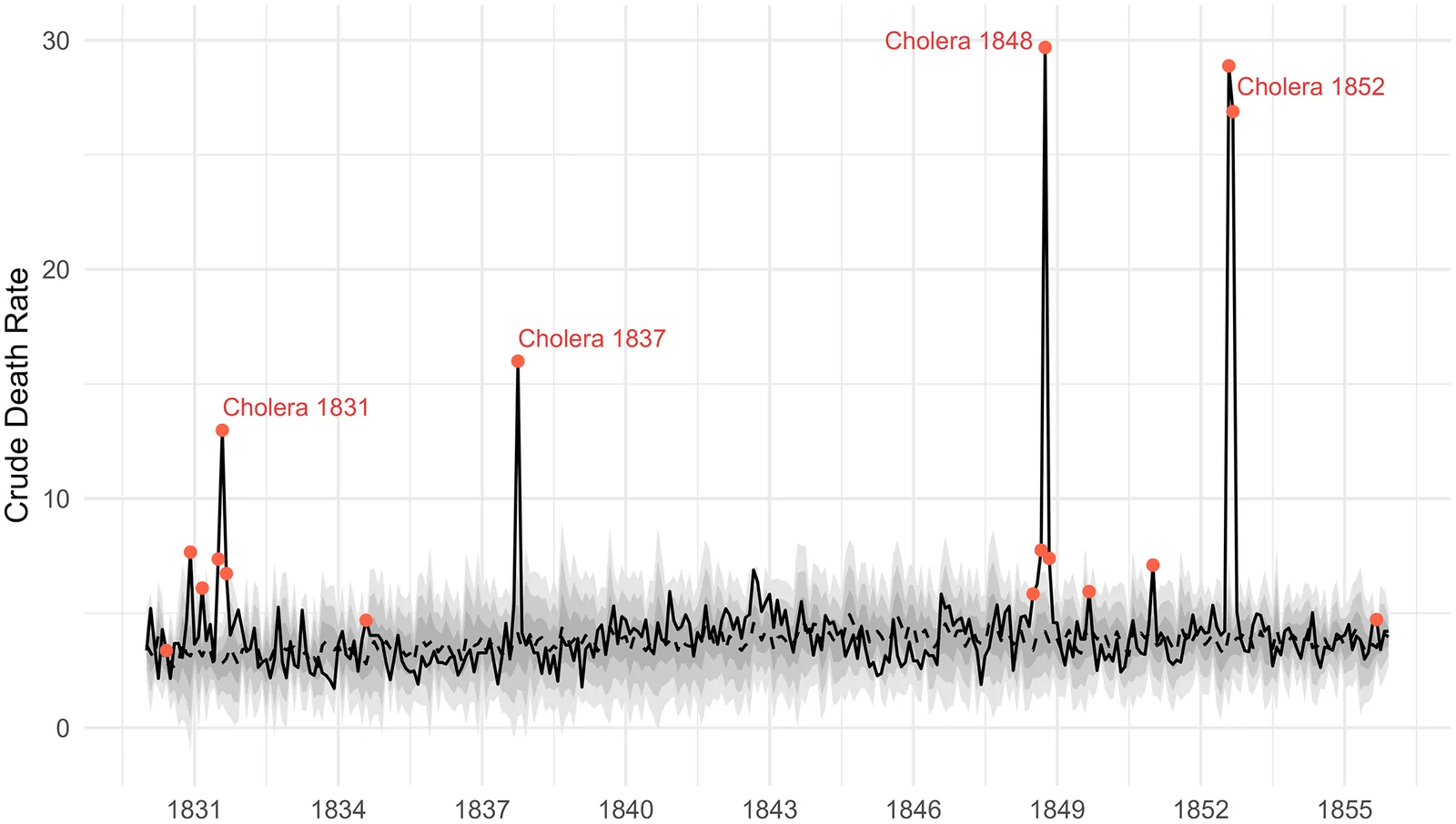
Monthly crude death rate in Poznań and observations with unusually high values, 1830–1855. Authors’ calculations based on the Poznań Historical Population Database. The solid line represents monthly crude death rates, the dashed line indicates the reference mean, and the grey shaded area represents the ± 3 standard deviation range. Red points indicate outliers, with major mortality peaks labelled according to documented cholera outbreaks.

Rye prices were derived from official price tabulations published in the *Allgemeine Preußische Staats-Zeitung* and its successor titles, as well as in the *Amtsblatt der Königlichen Regierung zu Posen* [66,67]. The Prussian authorities reported monthly prices for staple grains and potatoes; however, systematic missing values occurred between June 1851 and August 1853. Missing rye price values were imputed using a random forest algorithm. Prices from Warsaw, obtained from Stanisław Siegel’s work [68], were used as the training dataset. This approach was justified by the strong economic links between Poznań and Warsaw and increasing market integration in 19th-century Europe resulting from transportation improvements, growing commercial exchange, and efforts to harmonise trade standards [69] (Fig 5).

**Fig 5:**
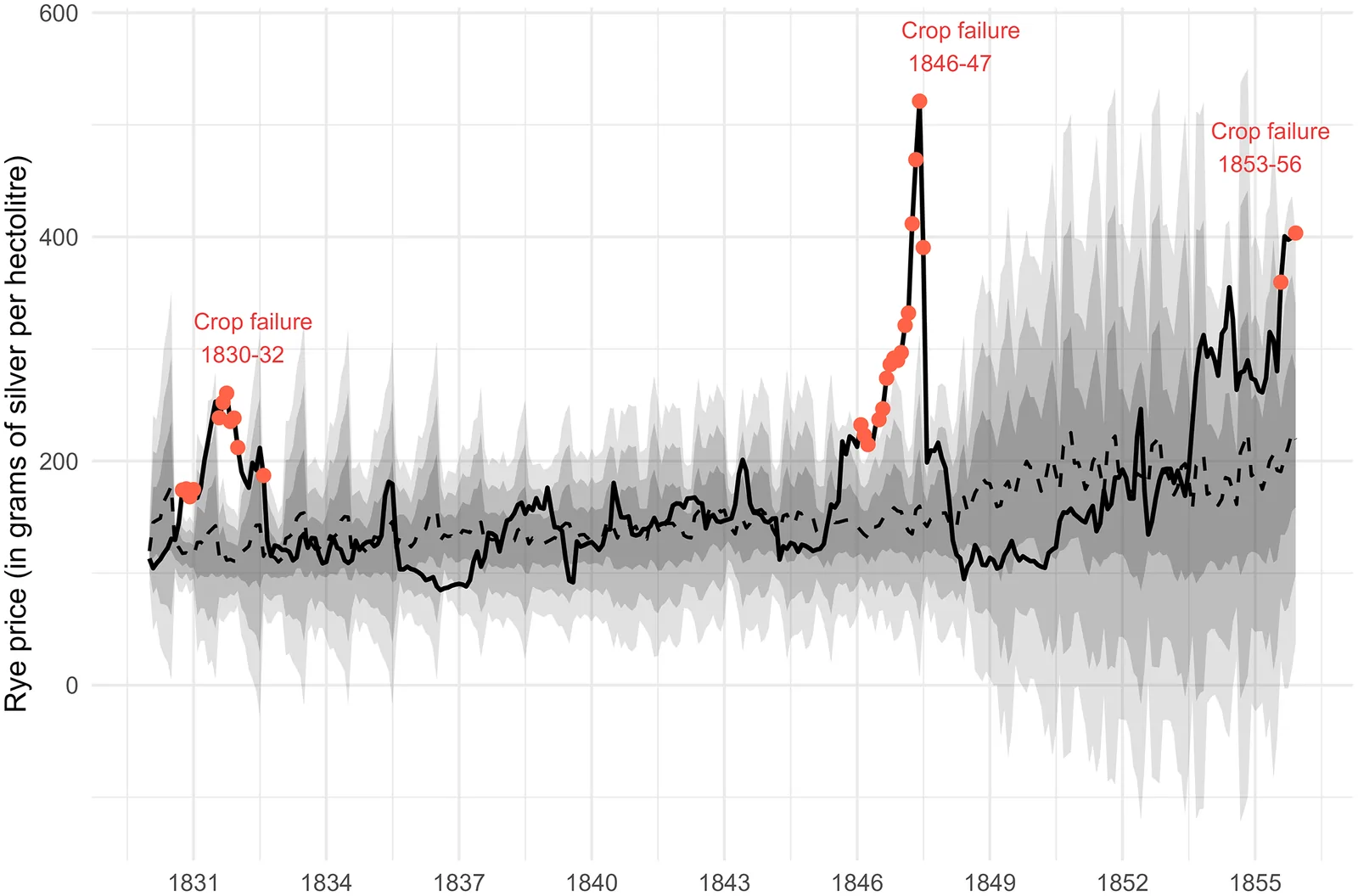
Monthly rye prices in Poznań and observations with unusually high values, 1830–1855. Authors’ calculations based on [66,67]. Data compiled and provided by Kalle Kappner. The solid line represents monthly rye prices, the dashed line indicates the reference mean, and the grey shaded area represents the ± 3 standard deviation range. Red points indicate outliers, with major peaks labelled according to documented crop failures.

Finally, flood exposure was identified separately because water-level measurements represent a different type of environmental indicator. Daily water-level observations from February to April for the years 1829–1855 were compiled from measurements of the Warta River at the Chwaliszewski Bridge in Poznań. These months were selected because they correspond to seasonal increases in water levels caused by spring snowmelt [70]. The seasonal distribution of water levels during the study period is presented in Fig 6. The flood threshold was set at 550 cm, as historical sources indicate that at this level a substantial part of Poznań was affected by flooding [71]. Daily measurements were used to identify specific high-water events exceeding the flood threshold (Fig 7).

**Fig 6:**
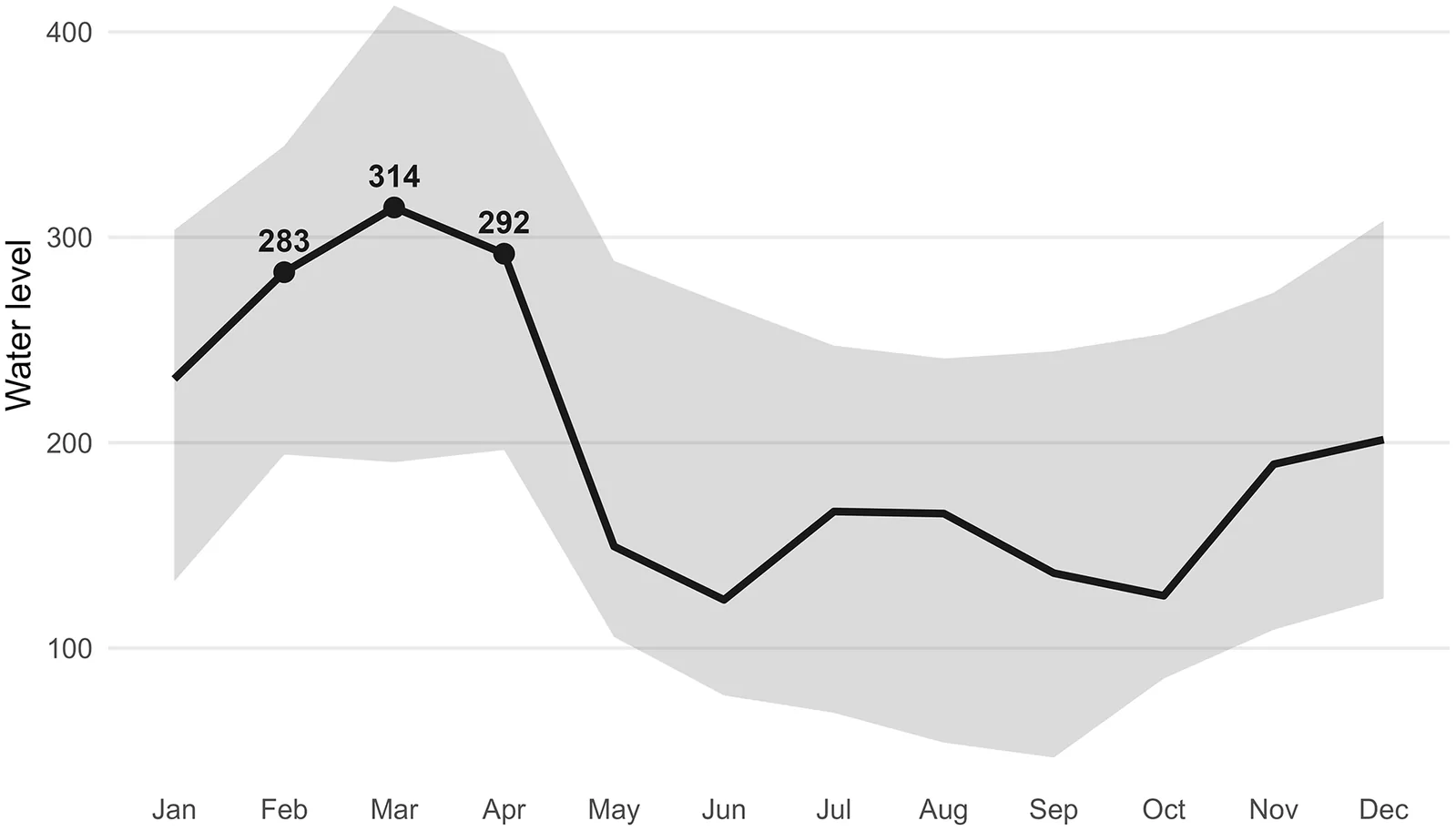
Monthly median water level (cm) of the Warta River at the Chwaliszewski Bridge, 1830–1855. Authors’ calculations based on data from the Archive of the Institute of Meteorology and Water Management – National Research Institute (IMGW-PIB), Poznań. The grey band represents the interquartile range.

**Fig 7:**
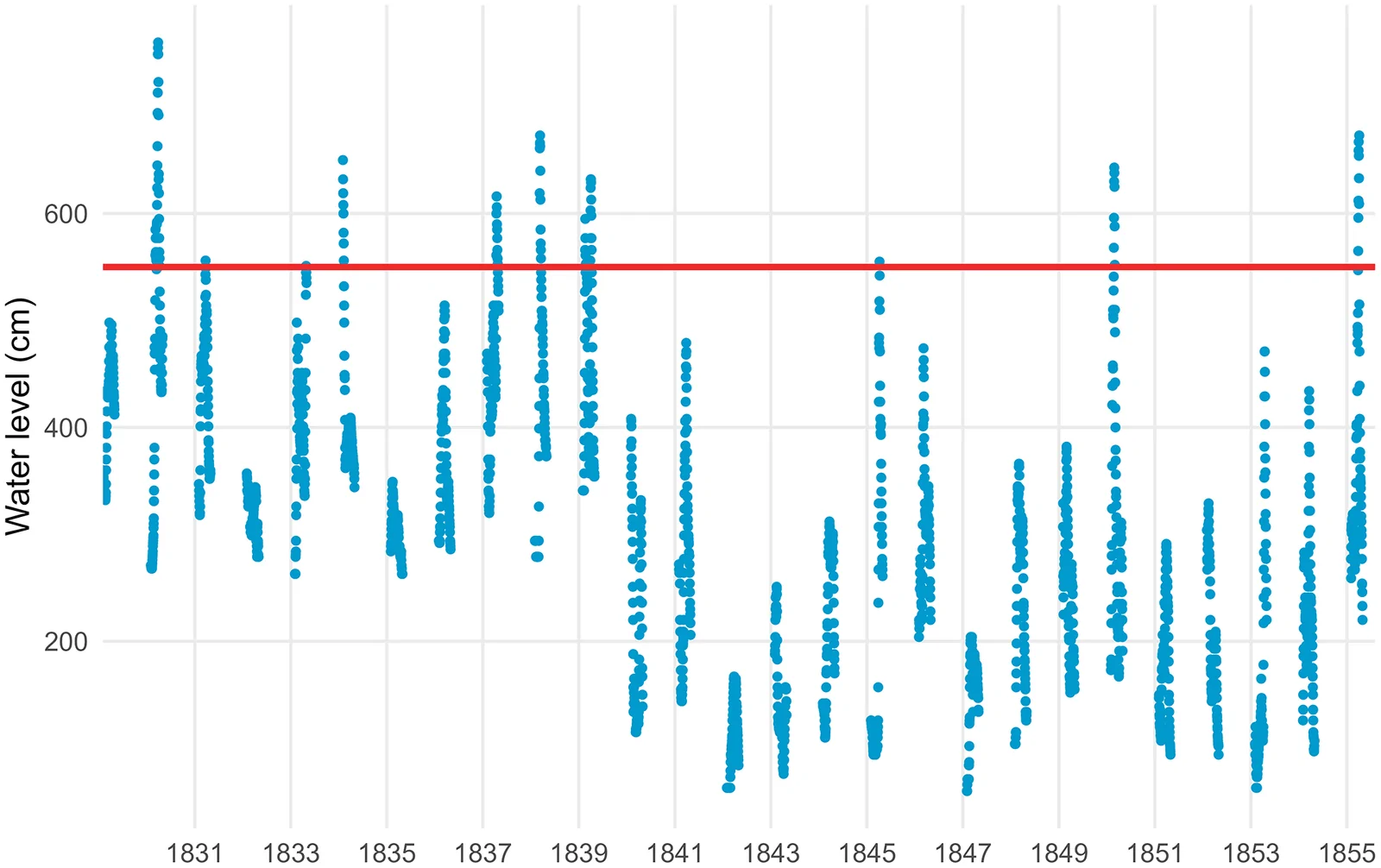
Daily water levels (cm) in February, March, and April, 1829–1855. Source: As in Fig. 6. The 550 cm threshold is indicated by a red line; each dot represents a single day.

### Statistical analysis

We used logistic regression models to assess the relationship between adverse early-life conditions and later-life social outcomes. Two logistic regression models were estimated. Model 1 examined the probability of entering a first marriage by age 45, and Model 2 examined socioeconomic mobility among individuals who entered marriage. The dependent variable in Model 1 was a binary indicator of whether an individual entered marriage during the observation period. The analytical sample included all individuals born between 1830 and 1855 who survived to at least age 15 (N = 15,121). The explanatory variables included indicators of early-life conditions during the prenatal period, infancy, and early childhood, as well as demographic and socioeconomic characteristics. The model included the following control variables: socioeconomic status at birth, sex, birth season, birthplace (Poznań or suburban village), religious denomination, and mother’s marital status at birth (Table 1).

**Table 1:** Description of the sample and the variables used in Model 1.

	N
*Sex*	
Men	7679
Women	7442
*SES at birth*	
Unskilled manual workers	6125
Farmers	839
Medium-high class	8157
*Birth place*	
Poznań	11544
Suburban village	3577
*Denomination*	
Catholics	11546
Lutherans	3575
*Birth season*	
Winter	4099
Spring	3862
Summer	3521
Autumn	3639
*Mother’s marital status*	
Single	450
Married	14671
*Stressors*	
Low SRB	2163
High rye price in the prenatal period	2495
Flood in the prenatal period	3520
High IMR	11644
High rye price in infancy	3795
High CDR (2–3 years of life)	8035
High rye price (2–3 years of life)	5466

The second logistic regression model examined whether exposure to adverse early-life conditions affected socioeconomic mobility among individuals who entered marriage. The dependent variable was the transition between parental socioeconomic status recorded at birth and the individual’s occupational status recorded at first marriage (Table 2). The analysis was restricted to individuals whose birth and marriage records could be linked successfully and whose occupational or social status was entered in the marriage record (N = 802). Because occupational information was available only for married individuals, Model 2 analyses socioeconomic mobility among individuals who entered marriage. Two binary outcomes were analysed separately: downward mobility and upward mobility. Downward mobility was defined as a transition from a higher parental occupational category to a lower occupational category, whereas upward mobility was defined as a transition from a lower parental occupational category to a higher occupational category.

**Table 2:** Description of the sample and the variables used in Model 2.

	N
*Sex*	
Men	297
Women	505
*SES at birth*	
Low	378
Medium-high	424
*Birth place*	
Poznań	400
Suburban village	402
*Denomination*	
Catholics	706
Lutherans	96
*Birth season*	
Winter	212
Spring	172
Summer	204
Autumn	214
*Stressors*	
Low SRB	81
High rye price in the prenatal period	127
Flood in the prenatal period	230
High IMR	616
High rye price in infancy	175
High CDR (2–3 years of life)	382
High rye price (2–3 years of life)	253

Social status at birth and at marriage was coded based on occupation using the SOCPO classification. To ensure sufficient sample sizes, occupational categories were aggregated differently depending on the analytical objective. For Model 1, occupations were grouped into three broader categories: a) low-skilled manual workers (SOCPO groups 1 and 2), b) peasant farmers (SOCPO group 41), c) medium and high social strata (SOCPO groups 3, 42, and 5). For Model 2, occupations were grouped into two categories to capture upward and downward mobility: a) low-skilled manual workers (SOCPO groups 1 and 2), b) medium and higher social strata (SOCPO groups 3, 41, 42, and 5). Social mobility was defined as a change between these occupational categories from parental status at birth to the individual’s occupational position recorded at first marriage.

As a robustness check for Model 2, we estimated Fine–Gray competing risks models (see S1 Table in S1 File the supplementary material). These models were used to evaluate whether accounting for competing risks affected the interpretation of socioeconomic mobility results.

To assess potential overadjustment, we re-estimated the models after excluding SRB, infant mortality, and CDR, which high rye prices could influence. The reduced specifications retained high rye price and flood exposures, as well as the baseline covariates (S2–S3 Tables in S1 File the supplementary material). All statistical analyses were conducted using the procedures described above.

## Results

The results of Model 1, which estimated the probability of entering a first marriage by age 45, are presented in Table 3. Exposure to several early-life conditions was associated with differences in the probability of entering marriage, although the effects varied depending on the timing and type of exposure. A low sex ratio at birth (SRB) in the year of conception, considered an indicator of adverse conditions, was associated with lower odds of entering marriage (OR = 0.716, p = 0.011). Similarly, exposure to high rye prices during infancy was associated with reduced odds of marriage (OR = 0.735, p = 0.008). In contrast, individuals exposed to high rye prices during the prenatal period had higher odds of entering marriage (OR = 1.49, p = 0.002). No statistically significant associations were observed for flood exposure during the prenatal period, high infant mortality rate during the first year of life, or high rye prices during ages 2–3 years. Exposure to high crude death rates during ages 2–3 years showed a negative association with marriage probability, although this effect did not reach statistical significance (OR = 0.863, p = 0.068). Among adverse conditions characteristics, individuals born into farming families had higher odds of entering marriage compared with individuals from low-skilled manual worker families (OR = 1.83, p < 0.001). Individuals from medium and high social strata had lower odds of marriage compared with the reference group (OR = 0.818, p = 0.018).

**Table 3:** Estimated odds ratios for factors influencing the probability of entering marriage.

	OR	p
*Stressors*		
None (ref.)	1	
Low SRB	0.716	**0.011**
High rye price in the prenatal period	1.49	**0.002**
Flood in the prenatal period	1.13	0.163
High IMR	1.01	0.933
High rye price in infancy	0.735	**0.008**
High CDR (2–3 years of life)	0.863	0.068
High rye price (2–3 years of life)	1.01	0.874
*SES at birth*		
Unskilled manual workers (ref.)	1	
Farmers	1.83	**<0.001**
Medium-high class	0.818	**0.018**
*Sex*		
Men (ref.)	1	
Women	1.84	**<0.001**
*Birth season*		
Winter (ref.)	1	
Spring	0.817	**0.046**
Summer	1.11	0.303
Autumn	1.04	0.668
*Birth place*		
Suburban village (ref.)	1	
Poznań	0.39	**<0.001**
*Denomination*		
Catholics (ref.)	1	
Lutherans	0.616	**<0.001**
*Mother’s marital status*		
Married (ref.)	1	
Single	0.017	**0.004**
N	15121	
Events	905	
Pseudo R² (McFadden)	0.084	

Statistically significant values are marked in bold.

Sex, birthplace, denomination, and mother’s marital status were also significantly associated with marriage probability. Women had higher odds of entering marriage than men (OR = 1.84, p < 0.001). Individuals born in Poznań had lower odds of entering marriage compared with those born in suburban villages (OR = 0.390, p < 0.001). Lutherans had lower odds of marriage compared with Catholics (OR = 0.616, p < 0.001). Individuals born to unmarried mothers had substantially lower odds of entering marriage compared with those whose mothers were married (OR = 0.017, p = 0.004). The final Model 1 included 15,121 individuals and 905 marriage events. The McFadden pseudo-R² was 0.084.

The results of Model 2, examining socioeconomic mobility between parental status at birth and occupational status at first marriage, are presented in Table 4. Overall, early-life exposure indicators showed limited associations with socioeconomic mobility among individuals who survived to adulthood and entered marriage. For downward mobility, exposure to high crude death rates during ages 2–3 years was associated with lower odds of downward mobility (OR = 0.509, p = 0.028). No statistically significant associations were observed for other early-life indicators, including low SRB, rye prices, flood exposure, or infant mortality. For upward mobility**,** birthplace was the only statistically significant predictor. Individuals born in Poznań had higher odds of upward mobility compared with individuals born in suburban villages (OR = 3.73, p < 0.001) and lower odds of downward mobility (OR = 0.220, p < 0.001). No statistically significant associations were observed between early-life adversity indicators and upward mobility. The downward mobility model included 424 individuals and 99 events, whereas the upward mobility model included 378 individuals and 174 events. McFadden pseudo-R² values were 0.136 and 0.117, respectively.

**Table 4:** Estimated odds ratios for factors influencing social mobility at first marriage.

	Downward mobility		Upward mobility	
	OR	p	OR	p
*Stressors*				
None (ref.)	1		1	
Low SRB	1.52	0.371	2.25	0.106
High rye price in the prenatal period	0.844	0.731	0.652	0.360
Flood in the prenatal period	0.790	0.453	0.716	0.242
High IMR	0.656	0.180	1.17	0.611
High rye price in infancy	1.12	0.804	1.24	0.593
High CDR (2–3 years of life)	0.509	**0.028**	0.885	0.662
High rye price (2–3 years of life)	0.809	0.506	1.21	0.510
*Sex*				
Men (ref.)	1.		1	
Women	0.652	0.094	0.812	0.373
*Birth season*				
Winter (ref.)	1		1	
Spring	0.723	0.357	1.45	0.271
Summer	0.911	0.793	0.917	0.786
Autumn	0.877	0.696	0.833	0.574
*Birth place*				
Suburban village (ref.)	1		1	
Poznań	0.22	**<0.001**	3.73	**<0.001**
*Denomination*				
Catholics (ref.)	1		1	
Lutherans	0.448	0.094	2.65	0.070
N	424		378	
Events	99		174	
Pseudo R² (McFadden)	0.136		0.117	

Statistically significant values are marked in bold.

Results from Fine–Gray competing risks models are presented in Supplementary Table S1 in S1 File. The robustness analyses produced results consistent with the logistic regression models, with the direction and statistical significance of the main associations remaining largely unchanged.

The sensitivity models (Tables S2–S3 in S1 File the supplementary material) produced broadly similar directional patterns, although the magnitude and precision of some estimates changed. Estimates for social mobility remained imprecise, and the substantive conclusions were unchanged.

## Discussion

### Early-life adversity, marriage formation, and selection mechanisms

Our results demonstrate that early-life conditions were associated with later-life demographic outcomes in 19th-century Poznań, although the strength and direction of these associations varied depending on the type and timing of exposure. The findings support the interpretation that early-life adversity operated through both scarring and selection mechanisms. On the one hand, the most vulnerable individuals were removed early in life (selection), while on the other, surviving children experienced lasting negative effects of adverse conditions (scarring), which in turn influenced their health and social status later in life [6,72]. The importance of selection is also highlighted by Almond and Currie [73], particularly in contexts of severe crises or poor health environments. Individuals conceived in years with a low sex ratio at birth, reflecting adverse prenatal conditions and maternal stress [74], may have been particularly susceptible to scarring effects. Poor conditions during the prenatal period could have compromised individuals’ health, which in turn limited their physical and social capacities as well as their attractiveness, thereby reducing the likelihood of marriage later in life [75]. At the same time, a selection mechanism was likely at work: due to miscarriages, stillbirths, and perinatal mortality, only the more resilient fetuses survived. Consequently, those who endured malnutrition or flood-related stress may have exhibited, on average, better health in adulthood [76].

Previous research indicates that in the 19th century, declines in living standards caused by high rye prices could temporarily reduce reproduction, resulting in lower birth rates [77,78]. This effect was particularly pronounced among mothers from lower socioeconomic groups, who, facing deteriorating living conditions, may have adopted fertility-limiting strategies. A similar, though smaller, effect was observed for rye prices in the year preceding birth. The underlying mechanism was most likely a decline in fertility and an increased incidence of miscarriages, reflecting the physiological response to malnutrition [6,77].

The effect observed in Model 1, whereby exposure to high rye prices during the prenatal period increased the likelihood of later marriage, may thus be the result of selective fetal mortality or selective reproduction [6]. Evidence for the former has been documented in the nineteenth-century population of Poznań and, more broadly, in Greater Poland [17,19,20]. Analysis of the sex ratio at birth in Poznań and in the towns and villages of the province showed that in urban areas and among unmarried mothers, the proportion of male births fell significantly below the biological norm, which has been interpreted as the elimination of weaker male fetuses under economic and social stress. Notably, the phenomenon of selection in response to high food prices during pregnancy was also observed in nineteenth-century Scania [6]. The question of selective reproduction in response to declining living standards in the nineteenth-century population of Poznań, however, remains largely unexplored.

Surprisingly, Model 1 did not show a statistically significant effect of exposure to a high infant mortality rate in the first year of life. Previous research has identified this indicator as one of the most important “barometers” of adverse early-life conditions, allowing the assessment of environmental impacts on individual health and development. A strong negative effect of this measure on adult mortality has been demonstrated in the population of southern Sweden in the eighteenth and nineteenth centuries [16,25]. Years with particularly high infant mortality were often marked by epidemics of smallpox and whooping cough [16,25], with exposure in the first year to the latter disease increasing the risk of death in adulthood [6]. Similarly, being born during a whooping cough epidemic or in a year with high infant mortality was associated with reduced social mobility among women from lower social strata [6]. In addition, being born in a year with high infant mortality diminished the chances of attaining a high social status by age 50 [5].

In contrast, in nineteenth-century Poznań, exposure to high rye prices in the first year of life appears to have had a greater impact than disease load measured by IMR. The frailest individuals may have been less likely to survive conditions of high infant mortality to marriageable age. On the other hand, high food prices plausibly capture household living standards in a sensitive developmental period. Malnutrition may have impaired physical growth and later productivity, thereby reducing marriage chances due to poorer physical condition and constrained socioeconomic attainment [14,37,38].

Taken together, the positive association between prenatal high rye price exposure and marriage and the negative associations observed for low SRB and high rye prices during infancy may be consistent with opposing selection and scarring processes. However, the present analysis was not designed to distinguish these mechanisms or quantify their relative contributions. Because the marriage models are conditional on survival to marriageable age and do not model age-specific mortality over the life course, we cannot determine whether selection and scarring offset one another. Moreover, the indicators capture different and partly overlapping dimensions and periods of early-life adversity, and their coefficients cannot therefore be combined into a single net estimate. Selection and scarring should consequently be regarded as plausible interpretations of the observed associations rather than directly identified mechanisms.

### Socioeconomic background and marriage formation

Socioeconomic characteristics were important predictors of marriage formation, demonstrating that entry into marriage in 19th-century Poznań was strongly embedded in social and economic structures. Covariates indicating social status suggest that being born into a family of well-off peasant farmers significantly increased the likelihood of marriage in adulthood compared with low-skilled manual workers. This result is unsurprising, as for full-owning peasants, maintaining and passing on the farm to offspring was a key goal, ensuring continuity of social status and providing security in old age [51]. Surprisingly, the model indicates lower chances of marriage for individuals from the middle and upper social strata in Poznań. This pattern may have resulted from several overlapping factors, including prolonged investment in education and professional development, delayed marriage decisions, higher expectations regarding partner suitability and material security, and the greater costs associated with establishing an independent household. Selective marriage strategies, migration for work or study, and competition for partners of comparable social status within particular age cohorts may have further restricted marriage opportunities in this group [48]. The result may also partly reflect the composition of the sample. Men of higher socioeconomic status tended to marry at later ages, whereas women were more likely to marry earlier [34]. Consequently, differences in the sex and age distribution of individuals within socioeconomic categories may have contributed to the unexpectedly lower estimated chances of marriage among the middle and upper strata.

A significant effect of being born in spring on the likelihood of marrying later is observed. This finding aligns with observations of shorter lifespans among individuals born during this period, which may reflect poorer maternal nutrition during pregnancy due to limited food availability in the pre-harvest months [79]. Living in Poznań was associated with a lower probability of entering marriage compared with suburban villages, likely because peasant farmers, who generally had higher marriage prospects, were concentrated in rural areas. Significant denominational differences were observed, with Lutherans having lower odds of entering marriage than Catholics. By contrast, sex differences were pronounced, with women being considerably more likely to marry than men. Being born illegitimate in Poznań between 1830 and 1855 was associated with a markedly lower likelihood of entering marriage. This finding is consistent with research showing that children born to unmarried women generally experienced poorer health and social outcomes [80–82] and occupied weaker positions in the marriage market [39,83,84]. Moreover, health influenced mate selection in nineteenth-century populations [38,40], supporting the hypothesis that stigmatised illegitimate children faced social exclusion.

### Early-life adversity and socioeconomic mobility among survivors

The results concerning socioeconomic mobility provide a different perspective from those concerning marriage formation. Among individuals who survived childhood and entered marriage, early-life adversity showed limited associations with occupational mobility. This lack of strong associations should not necessarily be interpreted as evidence that early-life adversity had no socioeconomic consequences. Rather, it reflects the selective nature of the analysed population. Individuals included in the mobility analysis had already survived childhood, remained in the study area, and could be successfully linked from birth to marriage records.

Such selection is particularly important in historical populations, where mortality before adulthood was high and marriage occurred relatively late. Individuals most affected by poor nutrition, disease, or socioeconomic disadvantage may have died before adulthood, migrated, or remained outside the marriage market. Consequently, the remaining population may have been relatively homogeneous in terms of health and social potential, reducing the observable influence of early-life adversity on socioeconomic mobility [6,72,73].

The association between exposure to high crude death rates during ages two and three and lower odds of downward mobility requires careful interpretation. At first glance, this finding appears inconsistent with the expectation that disease exposure and mortality crises should increase later-life disadvantage. However, the observed association may again reflect selective survival. Periods of high childhood mortality may have disproportionately affected more vulnerable children, whereas survivors may have represented a positively selected group with greater biological resilience [6,72,76]. Therefore, this result should not be interpreted as evidence that exposure to adverse disease environments improved socioeconomic outcomes, but rather as evidence that observed associations in historical populations reflect both the consequences of adversity and the characteristics of survivors.

The absence of clear effects of early-life adversity on upward mobility may also reflect the structural limitations of 19th-century social mobility. Although occupational change occurred, social advancement depended strongly on family background, inheritance, education, and local economic opportunities. Previous studies of historical populations have emphasised that individual mobility was embedded within broader institutional and family contexts, limiting the extent to which personal characteristics alone determined socioeconomic trajectories [45].

Birthplace was an important predictor of both marriage formation and socioeconomic mobility. Individuals born in Poznań had lower odds of entering marriage compared with those born in suburban villages. Still, they also had higher odds of upward mobility and lower odds of downward mobility among those who married. This pattern may be partly explained by the increasing proletarianisation of the rural population in Greater Poland during the nineteenth century, which may have limited opportunities for upward social mobility [85,86].

## Conclusions

This study examined the long-term consequences of adverse early-life conditions for marriage formation and socioeconomic mobility in nineteenth-century Poznań using linked individual-level demographic records from the Poznań Historical Population Database. The findings indicate that early-life adversity was associated with later-life outcomes, although the observed effects depended on the life-course process considered. Prenatal adversity, reflected by a low sex ratio at birth, and nutritional stress during infancy were associated with a lower probability of entering marriage. In contrast, high rye prices during the prenatal period were associated with a higher probability of marriage, which may reflect selective survival processes. Exposure to high infant mortality did not significantly affect the probability of marriage. Among individuals who survived to adulthood and entered marriage, early-life conditions showed limited associations with socioeconomic mobility. The association between exposure to high crude death rates during ages two and three and lower odds of downward mobility highlights the importance of selection processes in shaping observed adult outcomes. Rather than indicating beneficial effects of adversity, this pattern likely reflects the survival of individuals who were more resilient to adverse environments.

Overall, the study demonstrates that the consequences of early-life adversity in historical populations cannot be understood solely through direct biological effects. Later-life outcomes reflected the combined influence of biological vulnerability, demographic selection, and social structures operating across the life course. By examining marriage formation and socioeconomic mobility simultaneously, this study extends previous research focused mainly on health and mortality outcomes and highlights the importance of considering selective processes when interpreting long-term consequences of early-life conditions.

### Research limitations

This study has several limitations that should be considered when interpreting the findings. First, analyses of marriage formation and socioeconomic mobility are constrained by the selective nature of historical records. The individuals included in the analyses of social mobility are those who survived childhood, remained in the study area, and could be successfully linked from birth to marriage records. Therefore, the analysed populations represent selected groups of survivors and may not fully capture the consequences of early-life adversity among individuals who died before adulthood, migrated, or never entered marriage. This issue is particularly important in 19th-century populations, where childhood mortality was high and marriage occurred relatively late. The average age at first marriage in 19th-century Poznań was 27.8 years for men and 25.4 years for women [34], meaning that individuals included in the analyses had already passed through several stages of biological and social selection.

Second, the indicators of early-life adversity used in this study represent population-level measures rather than direct observations of individual experiences. Although these indicators are widely used in historical demography as proxies for early-life conditions [5,6,16], they may be subject to measurement uncertainty because individuals exposed to the same environmental conditions could have experienced different levels of adversity. The population of Poznań during the period under analysis was severely affected by cholera epidemics and the famine of 1847, and its inhabitants lived in generally poor conditions [53–55; 87–89]. These adverse circumstances may partly explain the relatively high numbers of individuals exposed to some of the indicators.

Third, some indicators used in the study reflect complex demographic and biological processes that cannot be attributed to a single mechanism. For example, changes in the sex ratio at birth may reflect prenatal stress and selective fetal mortality but may also be influenced by fertility behaviour and other demographic processes [18–24]. Similarly, changes in rye prices indicate broader economic conditions but do not directly reveal household food availability, nutritional intake, or coping strategies.

Fourth, although the study uses individual-level linked demographic data, the possibility of linkage errors cannot be entirely ruled out. The automated record-linkage procedure was followed by manual verification to minimise false matches; however, historical records may contain incomplete information, spelling variations, and missing observations that limit the accuracy of reconstruction.

Finally, the study focuses on 19th-century Poznań, a specific urban environment shaped by Prussian political structures, rapid population growth, social inequality, and recurrent disease outbreaks [53–55; 87–89]. Therefore, the findings should be generalised cautiously to other historical populations, particularly rural communities or societies with different marriage systems, social structures, and disease environments. Nevertheless, the study provides important evidence on how early-life conditions shaped later demographic and socioeconomic outcomes in an urban historical population.

## Supporting information

S1 File Robustness and sensitivity analyses. Contains Tables S1–S3 presenting additional analyses of marriage and intergenerational social mobility.
(DOCX)
