# Supplementary material for: Adverse early-life conditions and their impact on the marriage market and social mobility: The Poznań birth cohort, 1830–1855
Source: PLoS One. 2026 Sep 28;21(9):e0345394. doi: 10.1371/journal.pone.0345394 (PMC13618944; doi:10.1371/journal.pone.0345394)
Supplement: Supplementary file 1 [file pone.0345394.s001.docx]

**Supporting information: robustness check and sensitivity analysis**

Table S1. Estimated Fine–Gray competing risks model coefficients for factors influencing social mobility at first marriage

|  | Downward mobility | | Upward mobility | |
| --- | --- | --- | --- | --- |
|  | HR | p | HR | p |
| *Stressors* | | | | |
| None (ref.) | 1 |  | 1 |  |
| Low sex ratio | 1.37 | 0.481 | 1.47 | 0.262 |
| High rye price in the prenatal period | 0.865 | 0.753 | 0.871 | 0.641 |
| Flood in the prenatal period | 0.783 | 0.323 | 0.734 | 0.116 |
| High IMR | 0.705 | 0.203 | 1.21 | 0.375 |
| High rye price in infancy | 1.05 | 0.904 | 1.01 | 0.975 |
| High CDR (2–3 year of life) | 0.585 | **0.048** | 0.95 | 0.787 |
| High rye price (2–3 years of life) | 0.825 | 0.470 | 1.23 | 0.320 |
| *Sex* | | | | |
| Men (ref.) | 1. |  | 1 |  |
| Women | 0.816 | 0.336 | 1.14 | 0.427 |
| *Birth season* | | | | |
| Winter (ref.) | 1 |  | 1 |  |
| Spring | 0.777 | 0.383 | 1.26 | 0.283 |
| Summer | 0.926 | 0.800 | 1.01 | 0.959 |
| Autumn | 0.924 | 0.762 | 0.876 | 0.563 |
| *Birth place* | | | | |
| Suburban village (ref.) | 1 |  | 1 |  |
| Poznań | 0.256 | **<0.001** | 2.52 | **<0.001** |
| *Denomination* | | | | |
| Catholics (ref.) | 1 |  | 1 |  |
| Lutherans | 0.434 | 0.081 | 1.39 | 0.180 |
|  |  |  |  |  |
| N | 424 |  | 378 |  |
| Events | 99 |  | 174 |  |
| Pseudo R² (Cox–Snell; pseudo-LR) | 0.139 |  | 0.135 |  |
| pseudo-LR | 63.69 |  | 54.65 |  |
| Wald χ²(13), p | 65.03, <0.001 |  | 57.05, <0.001 |  |

Statistically significant values are marked in bold.

Table S2. Sensitivity model for factors influencing the probability of entering marriage

|  | OR | p |
| --- | --- | --- |
| *Stressors* | | |
| None (ref.) | 1 |  |
| High rye price in the prenatal period | 1.24 | 0.070 |
| Flood in the prenatal period | 1.22 | **0.020** |
| High rye price in infancy | 0.765 | **0.018** |
| High rye price (2–3 years of life) | 0.991 | 0.920 |
| *SES at birth* | | |
| Unskilled manual workers (ref.) | 1 |  |
| Farmers | 1.78 | **<0.001** |
| Medium-high class | 0.814 | **0.016** |
| *Sex* | | |
| Men (ref.) | 1 |  |
| Women | 1.83 | **<0.001** |
| *Birth season* | | |
| Winter (ref.) | 1 |  |
| Spring | 0.815 | **0.044** |
| Summer | 1.08 | 0.413 |
| Autumn | 1.03 | 0.780 |
| *Birth place* | | |
| Suburban village (ref.) | 1 |  |
| Poznań | 0.388 | **<0.001** |
| *Denomination* | | |
| Catholics (ref.) | 1 |  |
| Lutherans | 0.608 | **<0.001** |
| *Mother's marital status* | | |
| Married (ref.) | 1 |  |
| Single | 0.018 | **0.004** |
|  |  |  |
| N | 15121 |  |
| Events | 905 |  |
| Pseudo R² (McFadden) | 0.082 |  |

Table S3. Sensitivity model for factors influencing social mobility at first marriage

|  | Downward mobility | | Upward mobility | |
| --- | --- | --- | --- | --- |
|  | OR | p | OR | p |
| *Stressors* | | | | |
| None (ref.) | 1 |  | 1 |  |
| High rye price in the prenatal period | 0.887 | 0.784 | 0.896 | 0.792 |
| Flood in the prenatal period | 0.893 | 0.707 | 0.722 | 0.223 |
| High rye price in infancy | 0.85 | 0.715 | 1.10 | 0.802 |
| High rye price (2–3 years of life) | 0.978 | 0.941 | 1.27 | 0.390 |
| *Sex* | | | | |
| Men (ref.) | 1. |  | 1 |  |
| Women | 0.678 | 0.124 | 0.851 | 0.485 |
| *Birth season* | | | | |
| Winter (ref.) | 1 |  | 1 |  |
| Spring | 0.728 | 0.365 | 1.36 | 0.355 |
| Summer | 0.837 | 0.612 | 0.917 | 0.784 |
| Autumn | 0.822 | 0.554 | 0.831 | 0.564 |
| *Birth place* | | | | |
| Suburban village (ref.) | 1 |  | 1 |  |
| Poznań | 0.237 | **<0.001** | 3.61 | **<0.001** |
| *Denomination* | | | | |
| Catholics (ref.) | 1 |  | 1 |  |
| Lutherans | 0.426 | 0.079 | 2.86 | **0.048** |
|  |  |  |  |  |
| N | 424 |  | 378 |  |
| Events | 99 |  | 174 |  |
| Pseudo R² (McFadden) | 0.124 |  | 0.110 |  |
